# A survey of how biology researchers assess credibility when serving on grant and hiring committees

**DOI:** 10.7717/peerj.20502

**Published:** 2026-01-15

**Authors:** Iain Hrynaszkiewicz, Beruria Novich, James Harney, Ross Gray, Veronique Kiermer

**Affiliations:** PLOS, San Francisco, California, United States

**Keywords:** Research assessment, Survey data, Open science, Scholarly communication

## Abstract

Researchers who serve on grant review and hiring committees have to make decisions about the intrinsic value of research in short periods of time, and research impact metrics such Journal Impact Factor (JIF) exert undue influence on these decisions. Initiatives such as the Coalition for Advancing Research Assessment (CoARA) and the Declaration on Research Assessment (DORA) emphasize responsible use of quantitative metrics and avoidance of journal-based impact metrics for research assessment. Further, our previous qualitative research suggested that assessing credibility, or trustworthiness, of research is important to researchers not only when they seek to inform their own research but also in the context of research assessment committees. To confirm our findings from previous interviews in quantitative terms, we surveyed 485 biology researchers who have served on committees for grant review or hiring and promotion decisions, to understand how they assess the credibility of research outputs in these contexts. We found that concepts like credibility, trustworthiness, quality, and impact lack consistent definitions and interpretations by researchers, which had already been observed in our interviews. We also found that, in our sample, assessment of credibility is very important to the majority (90%, 95% CI [87–92%]) of researchers serving in these committees but fewer than half of participants are satisfied with their ability to assess credibility. This gap between importance of an assessment and satisfaction in the ability to conduct it was reflected in multiple aspects of credibility we tested, and it was greatest for researchers seeking to assess the integrity of research (such as identifying signs of fabrication, falsification, or plagiarism), and the suitability and completeness of research methods. Non-traditional research outputs associated with open science practices—research data, code, protocols, and preprints—are particularly hard for researchers to assess, despite the potential of Open Science practices to signal trustworthiness. A substantial proportion of participants (57% [52%, 61%] of participants) report using journal reputation and JIF to assess credibility of research articles and outputs, despite journal reputation and JIF being proxies for credibility that rely on characteristics of research outputs that are extrinsic, rather than intrinsic, to the output itself. While our results only describe the practices and perspectives of our sample, they may suggest opportunities to develop better guidance and better signals to support the evaluation of research credibility and trustworthiness—and ultimately support research assessment reform, away from the use of proxies for impact and towards assessing the intrinsic characteristics and values researchers see as important.

## Introduction

Initiatives such as the Coalition for Advancing Research Assessment (CoARA; https://coara.eu/) and the Declaration on Research Assessment (DORA; https://sfdora.org/) aim to reform research assessment practices to focus on intrinsic qualities of research rather than extrinsic qualities, such as journal and institution-based metrics. These initiatives have elements in common: recognizing a diverse array of research outputs and contributions; basing assessment on qualitative expert judgements; and ensuring responsible use of quantitative metrics—in particular avoiding journal-based metrics such as the Journal Impact Factor (JIF), and institutional rankings, to judge individual researchers or studies.

The JIF and other journal-level metrics based on citations have been widely criticized for their use in research assessment (Rijcke et al., 2015; Aksnes, Langfeldt & Wouters, 2019; Larivière & Gingras, 2009), in part because they focus on impact rather than credibility and do not convey intrinsic qualities of research articles and outputs (we call them “extrinsic proxies” in this study). Author and institution reputation are other extrinsic proxies which can reinforce inequities in the research ecosystem.

Yet, despite strong calls for action (Hicks et al., 2015; Wilsdon et al., 2015), extrinsic proxies continue to be widely used in research assessment contexts (Rice et al., 2020). One explanation is that most assessment exercises are conducted under time constraints, such that evaluators, using what is easily measurable, overly rely on extrinsic proxies (Moher et al., 2018; Bornmann & Marewski, 2019). As an alternative, there have been calls for signaling intrinsic qualities of research more clearly at the article level (Jamieson et al., 2019). These calls for reform highlight a need to characterize what should be assessed at the article level and to develop policies and tools to enable and facilitate this form of assessment.

Researchers routinely need to assess research outputs in different contexts and may use different criteria depending on the context. For example, when researchers are discovering new information to inform their research, they consistently recognize quality and trustworthiness (also characterized as reliability or cognitive authority) as prime criteria. Both quality and trustworthiness determinations are influenced by social and traditional cues, which center on perceptions about peer review and the reputation of the journal. When dealing with information outside of their own field of study, these associations are weaker, and researchers tend once again to default to metrics like JIF (Rieh, 2002; Tenopir et al., 2015, 2010; Thornley et al., 2015). In the absence of journal-related signals, when research is only available as preprints, cues related to information about open science content and independent verification of authors’ claims have been suggested as important factors for judging credibility (Soderberg, Errington & Nosek, 2020).

In a previous interview study (Harney et al., 2021) we sought to more precisely characterize researchers’ attitudes and desired outcomes (i) when they participate in research assessment exercises on committees (the “committee context”) compared to (ii) when they are conducting their own research (the “discovery context”). This current study builds on and seeks to further quantify a subset of the findings from these interviews.

In our interview study involving 52 cell biology researchers from the US, UK, and EU (Harney et al., 2021), we focused on understanding how researchers evaluate the impact and credibility (or trustworthiness) of research outputs in these two different contexts. We found that despite a strong focus on impact in research assessment committee guidelines, assessing both credibility and impact are important to researchers in the committee context. We also found that researchers find it challenging to evaluate credibility of individual outputs and commonly rely on journal-based proxies to do this. Researchers in our interviews noted using journal reputation to, for example, make assumptions about a journal’s approach to peer review to inform their judgements. While there can be value in this approach, journal reputation is fundamentally extrinsic to the output. Further, journal metrics, specifically JIF, have been found to be poor predictors of the quality of peer review of an individual manuscript (Severin et al., 2023).

Given the pervasiveness and limitations of using extrinsic proxies in research assessment, in the current study we sought to validate the findings from our interviews that suggested a need for better signals of the intrinsic qualities of research in research assessment contexts.

From our interviews, we generated three hypotheses to test in this study:

- Credibility is important in the committee context. Evaluating the credibility of a candidate’s research outputs is very important to the majority (>50%) of researchers in our sample.
- Needs around credibility are not very well satisfied. Fewer than half of researchers in our sample are very satisfied with their ability to assess the credibility of research in the committee context.
- Assessments of credibility do not use optimal methods. The majority (>50%) of researchers (>50%) in our sample use proxies to evaluate credibility that convey extrinsic characteristics of research outputs in the committee context (“extrinsic proxies”).

In our interviews (Harney et al., 2021) we also observed a lack of standardized definitions for credibility and impact, which was independently observed by Morales et al. (2021). While we had working definitions of credibility (reflecting the likelihood that the work is trustworthy, robust and reliable) and impact (reflecting the influence in academia or on society) researchers in our interviews often referred to concepts of credibility, quality, reputation, prestige and impact interchangeably and in circular ways. To address the problem of a lack of standardized and exclusive definitions for credibility, we constructed the survey to ask about different aspects of credibility that surfaced during our interviews.

## Materials and Methods

### Ethical approval

Prior to survey distribution our study protocol was reviewed by an independent ethics committee Pearl IRB, who determined the study was exempt from requiring IRB approval, according to FDA 21 CFR 56.104 and 45CFR46.104(b)(2).

### Survey design

The survey questions were derived from the output of our previous qualitative study, which consisted of 52 interviews with researchers (Harney et al., 2021). Because in our interviews we observed that different terminology was used by participants when talking about the same concepts, we formulated partially redundant questions using terminology used by researchers in the interviews that may refer to different aspects of research assessment (*e.g*., “evaluate if research is trustworthy or reliable” and “evaluate if research is credible”). The questions referred to assessment of research outputs, but as the interviews revealed a strong emphasis on research articles, we also included a question specifically looking at different types of research output (that is, comparing traditional publications with preprints, code, lab protocols and datasets).

In structuring the survey, we adapted jobs-to-be-done (JTBD) theory (Ulwick, 2017) to elicit data on (i) what participants are assessing, *i.e*., aspects of credibility and/or impact and (ii) what they are trying to accomplish during the assessment of a specific piece of research or research output, *i.e*., the goals or outcomes being pursued. An example of (i) is “Evaluate if research is reliable and trustworthy”; an example of (ii) is “Evaluate if conclusions are well supported”. With this outcome-driven approach, the survey queried both the importance of each task and the satisfaction of the participant in their ability to successfully complete this task. We’ve used this design previously (Hrynaszkiewicz, Harney & Cadwallader, 2021) because it allows an analysis of areas of opportunity for new tools, interventions, or solutions—where the importance of a task is high but satisfaction with the ability to conduct it is low.

We supplemented a standard JTBD approach with questions about researchers’ commonly used approaches (methods) for assessing credibility, to test if researchers may be subjectively satisfied with methods that are objectively ineffective for assessing the intrinsic qualities or credibility of research (*e.g*., JIF). The survey asked participants what methods they normally use to evaluate each of the six aspects of credibility we tested. The response options (Table 1) included methods that were frequently mentioned in our interviews with cell biology researchers in the earlier qualitative phase. These included three proxies for assessing credibility and/or impact that are extrinsic to the research output rather than intrinsic: use of author or lab reputation, use of journal reputation, and use of JIF.

**Table 1:** List of methods to assess aspects of credibility that were included in the survey (derived from our previous interview study).

Evaluation methods focused on intrinsic factors (“intrinsic proxies”)	Evaluation methods focused on extrinsic factors (“extrinsic proxies”)
Review research output Review research methods Review figures Consulted related publications Review open peer reviews Check for shared data or code Consult with colleagues Check for citation by others Confirm output is peer reviewed	Use author or lab reputation Use journal reputation Use journal impact factor

Although not strictly germane to the hypotheses, we included questions about approaches for assessing impact to ensure that impact considerations were not confounded with credibility judgments. We also collected free text comments. The survey was programmed in the Alchemer survey tool. After pretesting of the survey, it was deployed on May 26, 2022 and remained open until July 8, 2022. The survey instrument is available in Figshare (Hrynaszkiewicz & Novich, 2023).

### Participant recruitment

We recruited participants who had served on a grant review committee or on a hiring committee in the past 2 years. To recruit a larger cross-section of researchers than in our interviews (Harney et al., 2021), which focused on cell biology researchers, and to ensure an adequate sample size, we recruited active researchers in all sub-disciplines of biology, with a focus on laboratory-based sub-disciplines of biology. We recruited participants in all geographies, but we anticipated a bias in responses from the US, UK and EU due to the predominance of these regions in the email lists used. All interview participants in our previous qualitative study were located in these geographies. We used screening questions to exclude participants who did not fit the scope of the survey. Participants were disqualified if they:

- Selected an area of expertise other than Biology and Life Sciences
- Had not served on a grant review or hiring and/or promotion committee or both within the last 2 years

We recruited participants from multiple sources between May 26 and July 7, 2022, to maximize both the size and diversity of our sample:

- Email survey invitations to individual researchers
- Email to list of researchers in biology who have published with PLOS (sent to 176,931 emails)
- Email to purchased list of researchers from Clarivate who have not published with PLOS (sent to 14,741 emails)
- Social media posts
- Paid placement on ResearchGate

Requests were also sent to 50 organizations or listservs to distribute the survey *via* email and/or social channels (Table S1).

Our participants do not represent a random sample of the wider biological researcher population who have served on committees and conclusions from findings are likely indicative of the sample. However, the sample extends upon our previous interview study (Harney et al., 2021) by including participants from all sub-disciplines of biology. While not representative and despite the limitations in participant recruitment, this larger sample adds to the available evidence of research assessment behaviors in these research communities.

### Data analysis

Data analysis was conducted in Microsoft Excel and in R (R Core Team, 2024). Statistical analysis was primarily descriptive in nature. The analysis included only completed responses, where “complete” was defined as having answered all required questions.

In our analysis of how participants define “credible research” and specifically, which aspects participants viewed as most closely related to “credibility”, we used Spearman’s R from the *psych* (Revelle, 2024) package in R to examine correlations between each aspect of credibility assessment with the question about the importance of evaluating if research is “credible”. Spearman’s R was used to assess correlations due to the ordinality and non-continuous nature of the response data. We further supported this analysis with a Principal Components Analysis (PCA) to understand the influence of each aspect on research credibility.

In our analysis of the importance of assessing research outputs and credibility in the committee context as well as analysis of whether participants felt satisfied with their ability to assess credibility, we examined the percentage of the survey population who answered each aspect of the assessment questions with very or extremely important/satisfied.

We also examined differences in importance and satisfaction by segment (discipline, geography, career stage/experience, and type of committee). Comparisons of segment response proportions were conducted using a z-test with Bonferroni correction using the *expss* (Demin, 2022) package in R, with a significance level set at *p* < 0.05. The denominator for the Bonferroni correction was the number of tests conducted per segment, *i.e*., the number of tests in the committee segment. This analysis is included in the Supplemental Information file and in Figshare (Hrynaszkiewicz & Novich, 2023).

## Results

We received 485 completed survey responses. The anonymized survey dataset is available in Figshare (Hrynaszkiewicz & Novich, 2023). Due to our recruiting participants *via* our own email lists and requesting that third parties (Table S1) distribute the survey on our behalf to an unknown number of recipients, we are unable to calculate an overall response rate. Of the completed responses, 374 (77%) came from emails sent to PLOS email lists. Based on emails sent alone (176,931 unique emails) the approximate lower bound of the response rate for email-recruited participants who went on to complete the survey is 0.21%. Many of these 176,931 potential recipients will likely not have met the inclusion criteria, particularly having served on a relevant committee in the past two years. Further, some emails will have been undelivered emails, caught in spam filters, or sent to unmonitored email addresses. The actual response rate from eligible recipients will likely be well above 0.21%. See Table 2 for participant demographics.

**Table 2:** Participant demographics. Respondents that preferred not to answer questions on their demographics were excluded from the denominator when calculating percentage and excluded from any statistical analysis conducted in our study.

Demographic category	Number of respondents	% of respondents
*Total*		
All respondents	485	100%
*Country*		
UK or EU	207	43%
US	182	38%
Other country	96	20%
*Discipline*		
Cell biology	50	10%
Other lab biology	396	82%
All other biology	36	8%
Prefer not to answer	4	
*Experience/Career Stage (Years since “PhD or equivalent degree”)*		
0–10 years	53	11%
11–15 years	56	12%
16 or more years	373	77%
Prefer not to answer	3	
*Type of committee served on*		
Grant only	110	23%
Hiring and/or promotion only	89	18%
Grant and hiring and/or promotion	286	59%
*Gender*		
Man	294	69%
Woman	130	31%
Non-binary, Self-describe	2	<1%
Prefer not to answer	58	

### How researchers define credibility

We asked researchers about the importance of credibility, as well as six distinct aspects of credibility assessment derived from our interviews. We correlated the importance rating for each aspect of credibility assessment with the question about the importance of evaluating if research is “credible” to determine how participants define “credible research” and specifically, which aspects participants viewed as most closely related to “credibility” (Table 3).

**Table 3:** Bivariate correlations of the six distinct aspects of credibility assessment with “Evaluate if research is credible”. Correlations were assessed using Spearman’s R.

Correlation with “Evaluate if research is credible”	Spearman’s r	95% CI	*P* value
Evaluate if research is trustworthy or reliable	0.57	[0.51–0.63]	< 0.001
Evaluate if research was well designed	0.41	[0.34–0.48]	< 0.001
Evaluate if research outputs are transparent and shared openly	0.26	[0.17–0.34]	< 0.001
Evaluate if research was conducted ethically	0.39	[0.32–0.47]	< 0.001
Evaluate if research is fully reported	0.37	[0.29–0.45]	< 0.001
Evaluate if research is impactful	0.13	[0.04–0.21]	0.006

Most of the aspects’ importance ratings were moderately correlated with “research is credible”, but none were strongly correlated (Table 3), suggesting that participants’ definition of “credible” is both variable and complex across these aspects of assessment. Evaluating if research is impactful showed a particularly low correlation with credibility (Table 3). These results are supported by the PCA, whereby reducing dimensionality in the data to the first two principal components, demonstrated all other aspects apart from “impactful” explained 46% of the variance of the first component but in low individual amounts, whereas “impactful” explained 13.9% of the variance along the second component, suggesting “impactful” is likely viewed as a separate concept (Table S2 and Fig. S1).

### Assessment of research outputs and credibility in the committee context

We first sought to confirm that researchers do actively assess a candidate’s research outputs (publications, preprints, protocols, code, and data) as part of their service on a hiring and/or promotion or grant review committee. Eighty-two percent (95% CI [79–86%]) of participants said it is very or extremely important to assess a candidate’s research outputs in the committee context. However, only 56% [51%, 60%] of participants are very or extremely satisfied with their ability to assess a candidate’s research outputs using the resources currently available to them. Peer-reviewed publications are considered the most important type of output (rated as very or extremely important by 81% [77%, 84%] of participants). The four other types of outputs are rated as lower in importance. Evaluating if shared datasets are credible and if shared lab protocols are credible are very or extremely important to just over than half of the researchers (56% [52%, 61%] and 51% [47%, 56%], respectively). Evaluating if shared code is credible and if preprints are credible are very or extremely important to fewer than half of the researchers (41% [36%, 45%] and 40% [35%, 44%], respectively) (Table S3).

**Figure 1:**
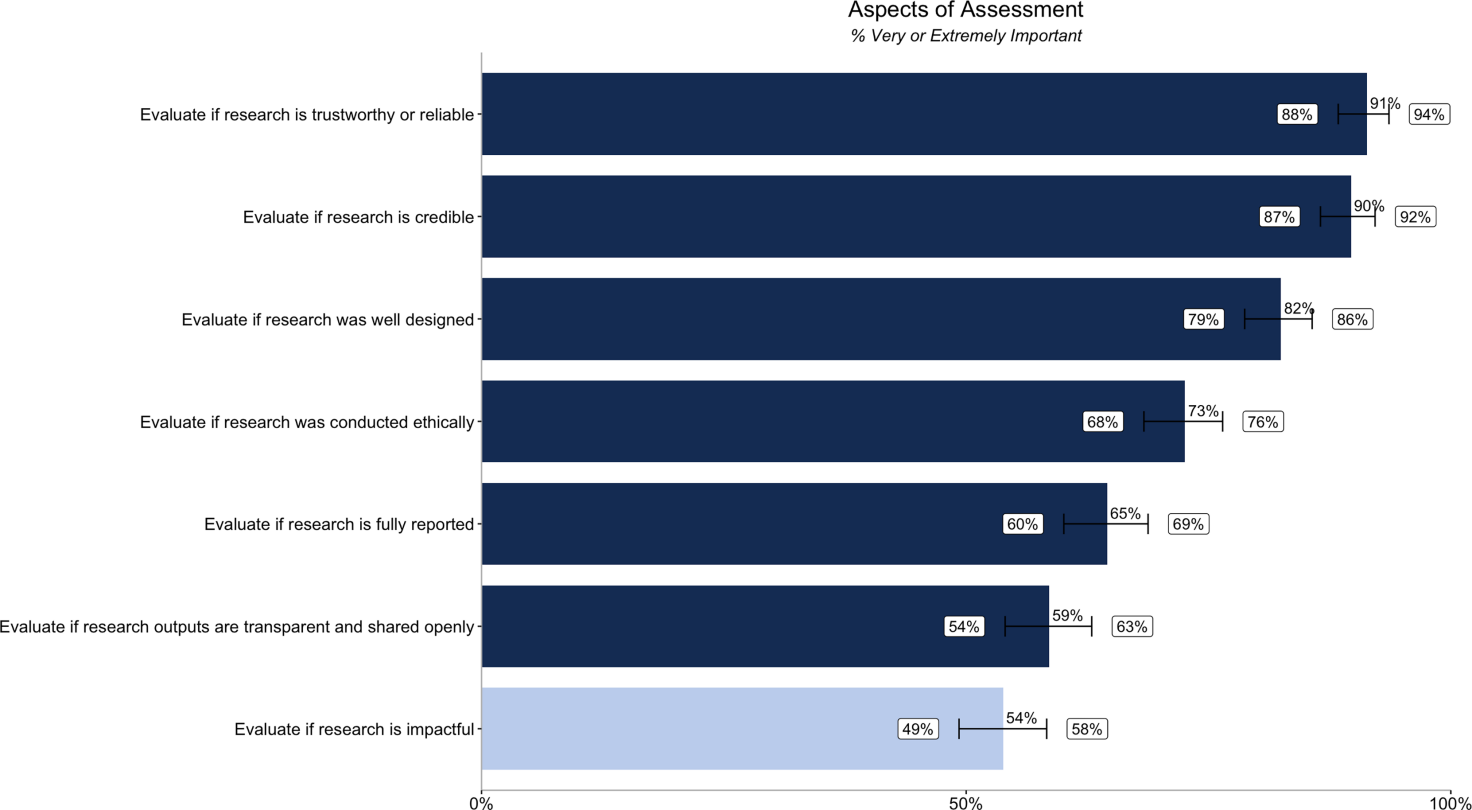
Percentage of respondents (*n* = 485) rating different aspects of research assessment as very or extremely important. Dark blue bars relate to the importance of assessing six different aspects of credibility, while the light blue bar relates to the importance of assessing impact. Error bars represent 95% confidence intervals.

We asked about the importance of assessing multiple aspects of assessment (six related to credibility, and one comparator question relating to impact) using terminology based on the previous interviews. When assessing research outputs, all aspects of credibility are very or extremely important to the majority (over 50%) of participants. The aspects of credibility rated highest in importance are evaluating if research is trustworthy or reliable (91% [88%, 94%]), credible (90% [87%, 92%]), and well designed (82% [79%, 86%]). Evaluating if research is impactful is less important (54% [49%, 58%]) than evaluating the various aspects of credibility (Fig. 1).

**Figure 2:**
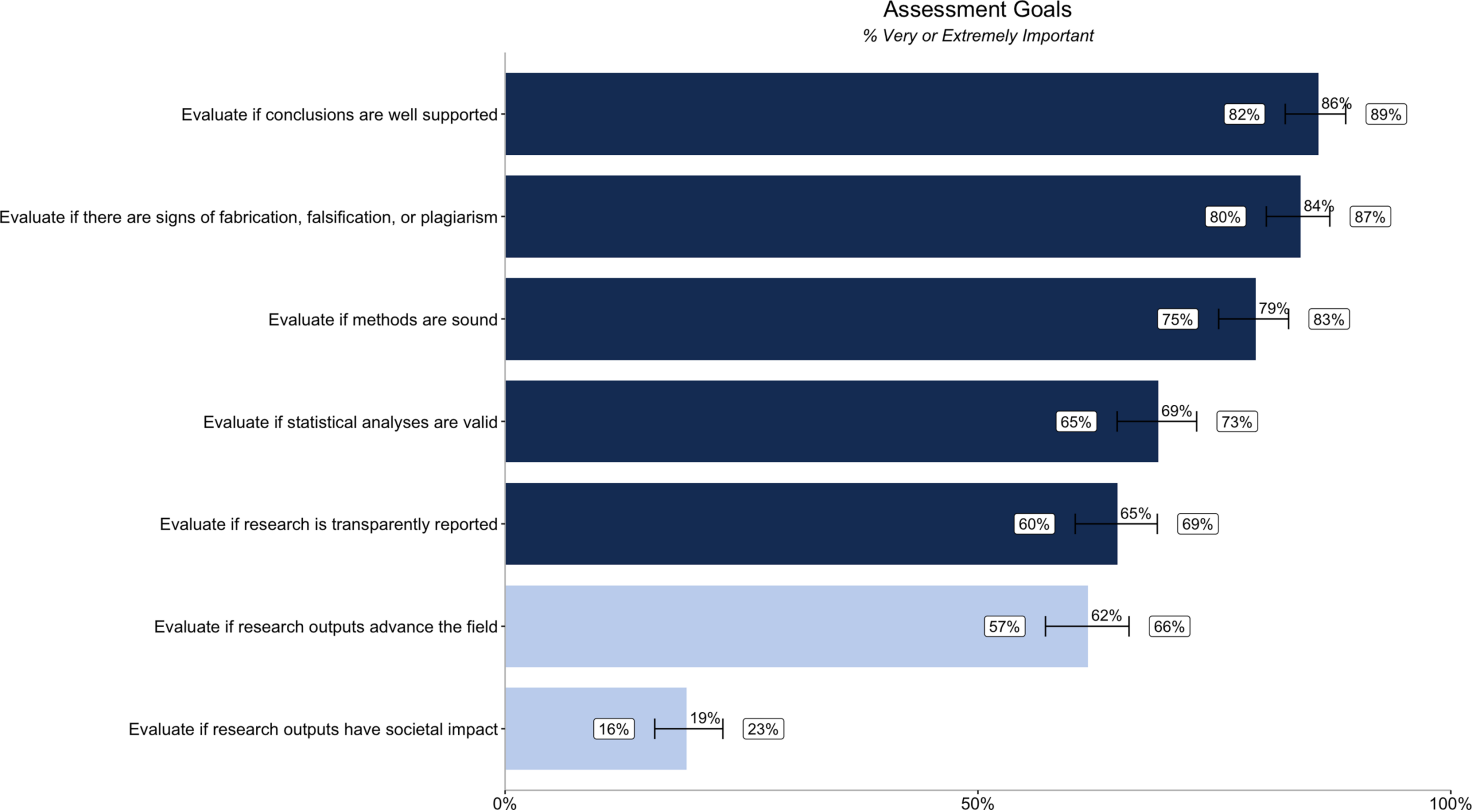
Percentage of respondents (*n* = 485) rating different research assessment goals as very or extremely important. Dark blue bars relate to the importance of credibility goals, while the light blue bars relate to the importance of assessing impact goals. Error bars represent 95% confidence intervals.

We also asked about credibility-related goals to understand what researchers are trying to accomplish—or the tasks they are trying to complete—when assessing credibility in the committee context. All the credibility-related goals we tested are considered very important by a majority of researchers when assessing a candidate’s research outputs. The most important goals are evaluating if conclusions are well supported (86% [82%, 89%]), if there are signs of fabrication, falsification, or plagiarism (84% [80%, 87%]), and if research methods are sound (79% [75%, 83%]). Only 19% [16%, 23%] believe it is very or extremely important to evaluate if research outputs have societal impact (Fig. 2). Differences in importance by segment (discipline, geography, career stage/experience, and type of committee) are reported in Table S4.

### Satisfaction with ability to assess credibility in committees

**Figure 3:**
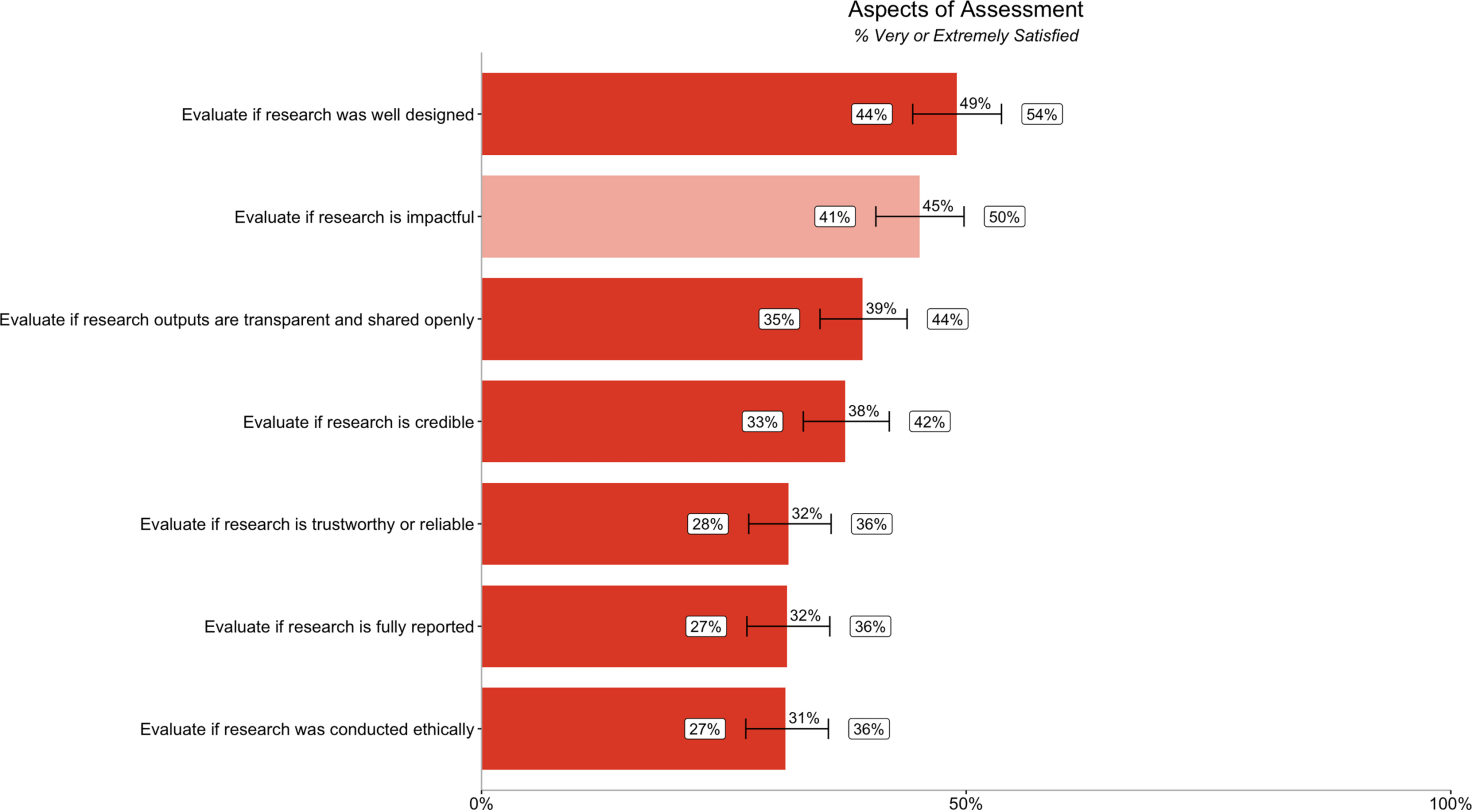
Percentage of respondents (*n* = 485) rating their ability to evaluate different aspects of research assessment as very or extremely satisfied. Dark orange bars relate to satisfaction with ability to evaluate six different aspects of credibility, while the light orange bar relates to satisfaction with ability to evaluate impact. Error bars represent 95% confidence intervals.

The majority of participants were not very satisfied with their ability to evaluate most aspects of credibility in committees that we asked about in our survey. Researchers are most satisfied with evaluating if research is well designed (49% [44%, 54%]). Researchers are least satisfied with evaluating if research was conducted ethically (31% [27%, 36%]), fully reported (32% [27%, 36%]), and trustworthy and reliable (32% [28%, 36%]). Researchers were more satisfied with evaluating impact (45% [41%, 50%]) than most aspects of credibility (Fig. 3).

**Figure 4:**
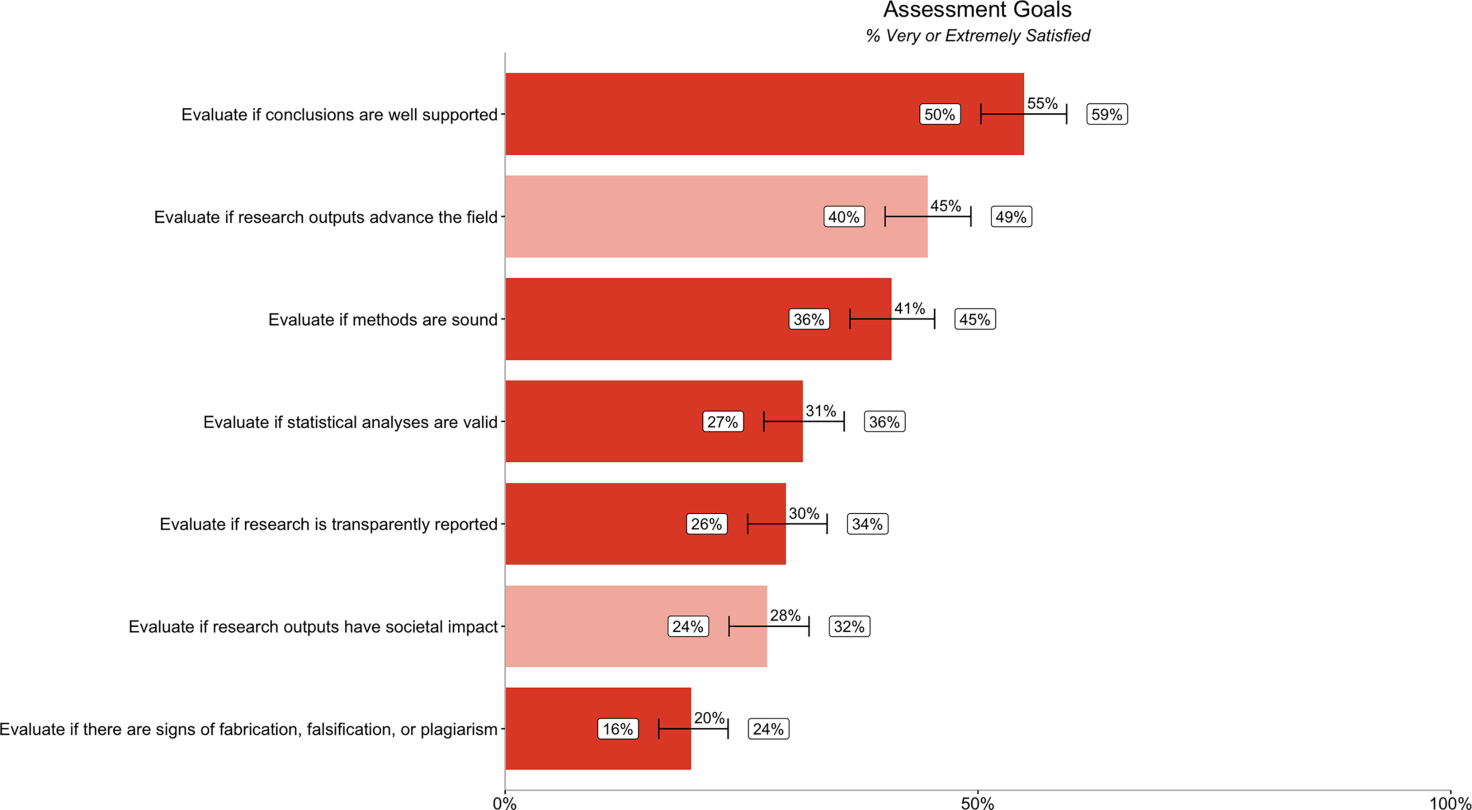
Percentage of respondents (*n* = 485) rating their ability to evaluate different research assessment goals as very or extremely satisfied. Dark orange bars relate to satisfaction with ability to evaluate credibility goals, while the light orange bars relate to satisfaction with ability to evaluate impact goals. Error bars represent 95% confidence intervals.

The low satisfaction with the ability to evaluate various aspects of credibility was mirrored in the answers to questions related to participants’ satisfaction with how well they could achieve stated goals to evaluate credibility (Fig. 4). ‘Evaluate if there are signs of fabrication, falsification or plagiarism’ showed the lowest level of satisfaction (20% [16%, 24%]). Fewer than half of researchers are very or extremely satisfied with their ability to achieve most of the credibility goals in our survey. However, slightly more than half of participants are very satisfied with their ability to evaluate if conclusions are well supported (55% [50%, 59%]). Differences in satisfaction by segment (discipline, geography, career stage/experience, and type of committee) are reported in Table S5.

### How credibility is assessed

The survey asked participants what methods they normally use to evaluate each of the six aspects of credibility we tested. Methods presented as response options are again derived from and expressed according to the terminology used by interviewed researchers in our previous study (Harney et al., 2021). These methods include reviewing figures, reviewing research methods, related publications, and outputs associated with open science practices, such as shared data, code, and peer reviews. These methods also included three proxies that are extrinsic to the research output including JIF (Table 1).

Slightly more than half (57% [52%, 61%]) of participants reported normally using at least one of the three extrinsic proxies (use author or lab reputation, use journal reputation, or use journal impact factor) to evaluate if “Research is credible”. However, for any given aspect of credibility, only 1–2% of participants rely exclusively on extrinsic proxies. The majority of participants report using personal inspection means—that is, reviewing research outputs, research methods, and figures—to evaluate almost all aspects of credibility we asked about in our survey. However, a relatively high percentage of researchers also report using the extrinsic proxy of journal reputation when evaluating research credibility (48% [43%, 52%] when evaluating if research is credible and 43% [39%, 48%] when evaluating if research is trustworthy or reliable). A smaller percentage report using JIF when evaluating different aspects of credibility (19% [15%, 22%] use it when evaluating if research is credible and 15% [12%, 19%] use it when evaluating if research is trustworthy or reliable) (Table S6). Differences in methods used by segment (discipline, geography, career stage/experience, and type of committee) are reported in Table S7.

### Identifying opportunities for improved solutions

We quantified the gap between importance (Fig. 1) and satisfaction (Fig. 3) ratings for participants’ ability to evaluate different aspects of assessment. We also quantified the gap between importance (Fig. 2) and satisfaction (Fig. 4) ratings for researchers’ goals when assessing credibility of research outputs (Table 4) as well as the gap between importance and satisfaction ratings for evaluating the credibility of different types of outputs. We then ranked the gap between importance and satisfaction (Table 4).

**Table 4:** Comparison of importance and satisfaction scores for respondents’ credibility assessment-related goals, ranked by size of the gap between importance and satisfaction. Importance or satisfaction based on very or extremely important/satisfied categories only.

Credibility related goals (When assessing a candidate’s research outputs, how important is it to you that you are able to…?/how satisfied are you with your ability to…?)	Importance (95% CI)	Satisfaction (95% CI)	Gap (Difference)
Evaluate if there are signs of fabrication, falsification, or plagiarism	84% [80–87%]	20% [16–24%]	64
Evaluate if research is trustworthy or reliable	91% [88–94%]	32% [28–36%]	60
Evaluate if research is credible	90% [87–92%]	38% [33–42%]	52
Evaluate if research was conducted ethically	73% [68–76%]	31% [27–36%]	41
Evaluate if methods are sound	79% [75–83%]	41% [36–45%]	39
Evaluate if statistical analyses are valid	69% [65–73%]	31% [27–36%]	38
Evaluate if peer-reviewed publications are credible	81% [77–84%]	46% [41–50%]	36
Evaluate if research is transparently reported	65% [60–69%]	30% [26–34%]	35
Evaluate if shared datasets are credible	56% [52–61%]	23% [19–27%]	34
Evaluate if research is fully reported	65% [60–69%]	32% [27–36%]	33
Evaluate if research was well designed	82% [79–86%]	49% [44–54%]	33
Evaluate if conclusions are well supported	86% [82–89%]	55% [50–59%]	31
Evaluate if shared lab protocols are credible	51% [47–56%]	25% [21–29%]	27
Evaluate if shared code is credible	41% [36–45%]	19% [15–23%]	22
Evaluate if research outputs are transparent and shared openly	59% [54–63%]	39% [35–44%]	19
Evaluate if research outputs advance the field	62% [57–66%]	45% [40–49%]	17
Evaluate if preprints are credible	40% [35–44%]	24% [20–28%]	15
Evaluate if research is impactful	54% [49–58%]	45% [41–50%]	9
Evaluate if research outputs have societal impact	19% [16–23%]	28% [24–32%]	−9

In areas with the largest gaps between importance and satisfaction we assume there is the greatest opportunity to provide new or better solutions to complete these tasks (Ulwick, 2017). The largest gaps relate to assessing the trustworthiness and integrity of research (whether there are signs of fabrication, falsification, or plagiarism). However, substantial gaps between importance and low satisfaction also exist in researchers’ ability to assess research design and methods, conclusions, ethical research conduct, and statistical analyses.

Regarding different types of research output, the results show that researchers were most satisfied with their ability to evaluate if peer reviewed publications are credible (46% [41%, 50%]). Other types of output—shared lab protocols, preprints, shared datasets, and shared code—are rated lower (only 19–25% very or extremely satisfied) and are also rated lower than peer-reviewed publications in terms of their importance (Table 4). The gap between importance and satisfaction is greatest for peer-reviewed publications, due to its importance relative to other outputs.

## Discussion

The qualitative study that preceded this survey (Harney et al., 2021) started from the premise that researchers evaluate research outputs in various circumstances and that these circumstances influence what they focus on and how they perform their assessment. Like others (Rijcke et al., 2015; Hicks et al., 2015; Moher et al., 2018; McKiernan et al., 2019), we have been concerned that committees for grant review or hiring and promotion decisions still focus too heavily on extrinsic factors, such as where research results are published, rather than intrinsic factors related to the quality of the research. We hypothesized that when researchers examine research outputs to inform their own research (which we’ve dubbed the “discovery” context), they may value credibility highly and may focus on intrinsic characteristics. Therefore, they may also consider these characteristics important and valuable, in principle, when they participate in committees for grant review or hiring and promotion decisions—even though in the “committee” context, they may not have the time, resources, or knowledge to apply the same scrutiny as in the context of their own research. By understanding what researchers find important in both contexts, and how satisfied they are with their ability to assess these important aspects, we hoped to identify opportunities to better characterize research outputs when they are disseminated, and ultimately facilitate assessments based on intrinsic characteristics.

The 52 interviews conducted during the qualitative phase indicated that credibility, or trustworthiness, which tended to be established based on personal examination of the research outputs, was very important in the “discovery” context and was also important in the “committee” context. In the present study, we support the notion that biology researchers who have participated in committees for grant review or hiring and promotion decisions in the past 2 years consider assessing the credibility of research outputs to be very important in a research assessment (“committee”) context. We tested different aspects of credibility, as articulated by researchers in our interviews, and all aspects were considered very or extremely important by the majority of participants, ranging from 59% to 91% (depending on the aspect). A smaller proportion of participants considered it very or extremely important to assess if research outputs are impactful (54% [49%, 58%]) or have a societal impact (19% [16%, 23%]). The small percentage of participants valuing societal impact may be reflective of our cohort being limited to laboratory biologists, who are less likely to work on applied research.

Our survey results also confirmed a lack of standardized definitions of concepts like credibility, trustworthiness, quality, and impact, which had already been observed in our interviews, Harney et al. (2021) and by others (Morales et al., 2021). While this was a challenge for our survey work, this lack of agreement on what credible research is also points to a broader problem for research assessment. It is hard to assess something that is ill-defined or interpreted in many different ways. However, our results validate that notions related to credibility are distinct from those related to impact and further suggest that different aspects of credibility and trustworthiness are valued differently by researchers. We propose that the aspects of credibility and the assessment goals that we confirmed to be important for researchers can be broken down into three categories: rigor, integrity, and transparency (Table 5).

**Table 5:** A proposed categorization of the aspects of credibility and the assessment goals we tested to inform a framework of research assessment criteria.

Category	Aspects of credibility and assessment goals tested in this study	% of respondent considering these items very or extremely important [95% CI]
Rigor	Conclusions are well supported	86% [82–89%]
	Research is well designed	82% [79–86%]
	Research methods are sound	79% [75–83%]
	Statistical analysis are valid	69% [65–73%]
Integrity	No signs of fabrication, falsification or plagiarism	84% [80–87%]
	Research is conducted ethically	73% [68–76%]
Transparency	Research is fully reported	65% [60–69%]
	Research is transparently reported	65% [60–69%]
	Research outputs are transparent and shared openly	59% [54–63%]

The rigor category includes questions such as whether the research is well designed, the research methods and statistical analysis sound and valid, and the conclusions well supported. This category tended to be considered very or extremely important by a large proportion of participants (69–86%) (Tables 4, 5). Similarly, the integrity category was also seen as very or extremely important by a large proportion of participants and included questions around the detection of signs of fabrication, falsification, or plagiarism (73–84%) (Tables 4, 5). Within this category, a smaller proportion of participants (73% [68%, 76%]) also saw questions related to the ethical conduct of research as very or extremely important. Finally, the transparency category included questions such as whether the research was fully or transparently reported. A smaller percentage of participants (59–65%) considered these aspects as very or extremely important.

In terms of transparency, we were particularly interested in the extent to which participants examined the research outputs associated with open science practices. Indeed, our previous interviews revealed that open science practices sometimes form part of credibility judgements (Harney et al., 2021). However, our survey results highlight the dominant importance of peer-reviewed publications in research assessment, over other outputs such as datasets, codes, protocols, and preprints. This is consistent with previous research concluding that peer review is a central criterion in determining whether people engage with scholarly content (Tenopir et al., 2015, 2010). While 81% [77%, 84%] of participants consider it very or extremely important to examine peer reviewed articles, examining open science outputs is seen as very important for a smaller percentage of participants. Fifty one percent [47%, 56%] of participants considered it very or extremely important to examine protocols, 56% [52%, 61%] to examine datasets, and a smaller percentage to examine code (41% [36%, 45%]) and preprints (40% [35%, 44%]). This lower level of engagement may be encouraged by the lack of visibility of open science practices in academic job descriptions and hiring processes (Khan et al., 2021) and by the lack of consistency among publishers in whether and how these outputs are made accessible alongside published articles (Cabanac, Oikonomidi & Boutron, 2021; Kim et al., 2020). However, our survey did not ask whether the availability of these research outputs influenced considerations around transparency. Soderberg, Errington & Nosek (2020) have previously found that signals of open science practices are associated with increased trustworthiness of preprints. Our results are compatible with the interpretation that open science practices—and the resulting availability of open research outputs like protocols, data, or code—might be signals of transparency that positively affect trustworthiness.

Our results demonstrate that, in our sample, researchers participating in committees for grant review or hiring and promotion decisions place more importance on assessing the credibility of the applicant’s research outputs than what could be inferred from documented tenure and promotion committee guidance (Rice et al., 2020; McKiernan et al., 2019; Khan et al., 2021; Niles et al., 2020). This suggests that the attitudes and motivations of assessors are not a barrier to strengthening the importance of credibility, relative to impact, in tenure and promotion committee guidance documents. As researchers have complex and variable definitions of research quality (as it refers to both credibility and impact), it is likely that no single characterization of research quality will be sufficient in all contexts. The importance of different aspects will depend on the context of assessment and what is valued by the individuals and institutions concerned. There is an opportunity to more precisely craft the language used in instructions to committee members to describe the aspects of research assessment that matter to the institution for whom the assessment is being conducted. We suggest that aspects related to rigor, integrity, transparency, and impact of research outputs are all important and may constitute an initial framework for research assessment criteria. Each criterion will need to be precisely defined in unambiguous terms, which should be informed by further research and should reflect the epistemic diversity of different research traditions (Leonelli, 2022).

Our approach to the survey instrument focused not only on the importance of assessing different aspects of credibility in the committee context but also on the level of satisfaction that researchers experience while completing these assessments. This approach offers insight into opportunities for new solutions that would improve research assessment.

With the exception of “Evaluate if research is well designed” (49% [44%, 54%]) and “Evaluate if conclusions are well supported” (55% [50%, 59%]), the majority of participants in our survey (>50%, when considering 95% confidence intervals) were not very satisfied with their ability to evaluate all aspects of credibility (Figs. 3, 4). Even researchers who would prefer not to might often have to rely on extrinsic proxies, due to a lack of better options. While researchers in our sample reported using extrinsic proxies alongside other methods, their prevalence in research assessment contexts indicates that the other methods alone aren’t fulfilling evaluation needs. While a smaller percentage report using JIF in particular, 19% [15%, 22%] do use it when evaluating if research is credible and 15% [12%, 19%] use it when evaluating if research is trustworthy or reliable (Table S6). Moreover, the real number may be higher, as many researchers may be hesitant to admit relying on JIF due to social desirability bias.

Our results show several credibility-related goals, or aspects of assessment, for which a large proportion of participants reported finding it very or extremely important to assess credibility, but only a small proportion reported being satisfied with their ability to conduct this assessment (Table 4). These may be areas of opportunity to develop new signals that can be used by researchers in research assessment settings. Importantly, our previous qualitative research highlighted that such solutions should not add to the time burden of performing these assessments (Harney et al., 2021).

For example, the opportunity seems particularly substantial for aspects of credibility in the integrity category (*e.g*., “Evaluate if there are signs of fabrication, falsification, or plagiarism”; “Evaluate if research was conducted ethically”). These results may reflect recent scandals in the peer-reviewed literature such as image manipulation (Shen, 2020), systematic manipulation of the publication process, predatory publishing, and paper mills (Systematic manipulation of the publication process, 2018). This finding is novel and suggests that those assessing research now need additional signals of credibility beyond a simple assurance that outputs have been peer reviewed. Publishers and other organizations (like preprint servers) that already conduct specific checks for some of these integrity questions could signal the results of these checks more explicitly at the article level in ways that are at least as convenient as the extrinsic proxies currently serving this function, such that researchers would be intrinsically motivated to adopt them, in time-constrained research assessment contexts.

With the support of automated technologies—including artificial intelligence (AI), machine learning and natural language processing—it is now possible to efficiently evaluate certain characteristics related to transparency. An example is the detection of open science practices in publications—an approach PLOS began to apply to all its publications *via* its Open Science Indicators project (Hrynaszkiewicz & Kiermer, 2022). Similarly, others have automated the evaluation of compliance with reporting guidelines (Schulz et al., 2022). Because of this progress in automation and because the principles of transparency apply broadly across scientific fields, signals of transparency have become feasible. Further research could explore the extent to which signals of transparency obtained through these automated processes improve assessment of credibility.

Rigor signals are more complex to define. Many operational elements of research rigor vary by field, by discipline, by scientific question, and even by measurement technique. Optimizing research rigor therefore requires deep technical knowledge and change strategies must address norms so granular that few outside a discipline (or sub-discipline) may be familiar with them. While an opportunity exists to provide signals of rigor to support research assessment on intrinsic characteristics, the definition and implementation of these signals remains more elusive.

### Limitations

It is not clear that our findings are applicable beyond the context of grant review and hiring and/or promotion committees in the biological sciences, or in the wider population of researchers outside our sample. Our findings build upon 52 interviews with researchers and attempt to quantify a subset of the findings from those interviews in a larger group of researchers, but participants may not be representative of all biology researchers who have served on committees, due to the convenience sampling approach used to recruit participants. Other disciplines may differ significantly. For example, one would expect far more emphasis on societal impact in public health, and researchers may not have the same views on importance and satisfaction when assessing research outside of the committee context. We could not recruit as many participants as we would have liked, which limited the size of subgroups for comparing segments. We had a high percentage of PLOS-affiliated participants (on PLOS lists as author, reviewer, editor, or interested researcher). Responses of PLOS affiliates to questions related to importance did not differ from those unaffiliated with PLOS but the former tended to rate satisfaction higher, though this difference was statistically significant for only one question.

Social desirability bias is another potential limitation (Grimm, 2010). Participants may have been influenced throughout the survey if they thought that they should be more invested in the importance of evaluating credibility or in using methods for doing so—many, for instance, may have underplayed how much they use extrinsic proxies, in particular JIF.

The reported lack of standardized definitions of concepts like credibility, trustworthiness, quality, and impact—observed in our own research and by others (Morales et al., 2021)—has been a particular challenge for the survey work. To mitigate this challenge, we built redundancy into our survey—asking about credibility using different aspects and goals expressed by researchers during the qualitative phase. However, we cannot rule out that participants brought their own interpretations and emphasized different elements in their answers.

## Conclusions

We found that assessing credibility of a candidate’s research outputs in research assessment committees (hiring, promotion and grant review) is very important to the majority of researchers (82% [79%, 86%]) in our sample. We found that researchers’ needs around assessing credibility, understood in terms of their ability to achieve their goals, are not very well satisfied. Fewer than half of participants were satisfied with their ability to assess credibility, for all but two of 11 aspects of credibility (“evaluate if research is well designed”; “evaluate if conclusions are well supported”) we asked about in our survey. Finally, we found that assessments of credibility do not use optimal methods, with the majority of participants in our survey (57% [52%, 61%]) using at least one proxy related to extrinsic characteristics (such as author or lab reputation, journal reputation, or JIF) to evaluate if research is credible—a practice research assessment reformers consider problematic. Our results also confirmed that, among participants in our survey at least, researchers have complex and overlapping definitions of research quality and credibility, as previously concluded by Morales et al. (2021).

These observations suggest opportunities to improve research assessment processes. Guidance provided to assessors may be improved by adopting clear, unambiguous definitions of the aspects of research quality that are of importance for the institution seeking the assessment. As many have already stressed, the reductive approach of using a single metric or a small set of metrics is inadequate to assess research and researcher quality, and it is dangerous in contexts where time pressure allows simple metrics to dominate judgment. Further, better signals of whether a piece of research meets diverse, specific, and intrinsic definitions of quality would likely improve research assessment. Our results suggest that there is a large area of opportunity to provide signals of credibility and trustworthiness. The specific aspects of rigor, integrity, and transparency we tested may inform the development of such signals. We infer that they would likely be welcomed by researchers participating in research assessment committees, because our participants consider credibility and trustworthiness very important and are dissatisfied with the current means of assessing these qualities at their disposal. The importance of credibility and trustworthiness in research assessment mirrors their importance when researchers examine work to inform their own research, and these novel signals would likely find uses in both contexts. However, any new signal would need to be convenient and easy to use if they are to supplant the pervasive use of extrinsic proxies such as JIF, journal, lab, or author reputation in research assessment. Future research could seek to further define and test these signals for their ability to meet assessors’ needs and improve research assessment processes.

## Supplemental Information

10.7717/peerj.20502/supp-1 Supplemental Information 1 Supplementary tables and figure. Supplementary Table 1-Organizations List of organizations contacted to request distribution of the survey. Supplementary Table 2-PCA Table Loadings. Principal Components Analysis showing the summary importance of each aspect of credibility assessment to the principal components as well as the table loadings. PCA was based on a correlation matrix. Supplementary Figure 1-PCA Analysis PCA Analysis showing the contribution of each aspect of credibility assessment to the first and second principal components. Colour indicates the level of contribution. Supplementary Table 3 -Ratings Importance and satisfaction ratings for participant s’ ability to assess aspects of assessment; different types of research outputs; and to achieve assessment-related goals. 95% confidence intervals are included in square brackets. Supplementary Table 4 -Importance by Segment Segmentation of importance ratings in Supplementary Table 3-Ratings by type of committee; discipline; country; career stage. Statistically significant (p < 0.05) differences between segments are indicated. Supplementary Table 5 -Satisfaction by Segment Segmentation of satisfaction ratings in Supplementary Table 3-Ratings by type of committee; discipline; country; career stage. Statistically significant (p < 0.05) differences between segments are indicated. Supplementary Table 6 -Methods Frequency of use of different methods for assessing the 7 different aspects of assessment, including a summary table to quantity use of “extrinsic proxies” (author or lab reputation, journal reputation, journal impact factor). 95% confidence intervals are included in square brackets. Supplementary Table 7 -Methods by Segment Segmentation of use of different methods in Supplementary Table 6-Methods by type of committee; discipline; country; career stage. Statistically significant (p < 0.05) differences between segments are indicated.

## Additional Information and Declarations

### Data Availability

The following information was supplied regarding data availability:

The raw data is available at Figshare: Hrynaszkiewicz, Iain; Novich, Beruria (2023). Dataset from: A survey of how biology researchers assess credibility when serving on grant and hiring committees. figshare. Dataset. https://doi.org/10.6084/m9.figshare.22522372.v3.
